# Leadless pacemaker implantation in superobese patient

**DOI:** 10.31083/j.rcm2304125

**Published:** 2022-04-02

**Authors:** Michele Malagù, Emanuele D’Aniello, Francesco Vitali, Cristina Balla, Vincenzo Gasbarro, Matteo Bertini

**Affiliations:** ^1^Cardiology Unit, Sant’Anna Hospital, University of Ferrara, 44124 Ferrara, Italy; ^2^Vascular surgery Unit, Sant’Anna Hospital, University of Ferrara, 44124 Ferrara, Italy

**Keywords:** Leadless pacemaker, Super obesity, Vascular surgery, Fluoroscopy

## Abstract

**Background:**

A 70 years-old superobese man (weighted 230 kg) was referred to our hospital for recurrent syncope due to asystole alternating to atrial fibrillation. Convectional pacing was highly challenging; therefore, it was decided to implant a leadless pacemaker in a multidisciplinary intervention with surgical management of the femoral venous access.

**Methods:**

In a fully equipped operating room with bariatric table and appropriately dimensioned fluoroscope, a vascular surgeon performed surgical isolation of the right common femoral vein. After that, we proceeded to insert sheaths via the femoral vein, and through that a steerable transcatheter delivery system for the device.

**Results:**

The implant was successful without complication.

**Conclusions:**

Leadless pacemaker implantation can be effectively and safely performed even in superobese patients. Vascular access, fluoroscopic guidance and electronic interrogation could be easily managed and do not constitute a limit.

## 1. Introduction

The Micra leadless pacemaker (Medtronic, Minneapolis, MN, USA) is currently used in clinical practice for the treatment of bradyarrhythmia all around the world, with good safety and efficacy performance [1]. The device is implanted in the right ventricle through a femoral vein with the use of a large introducer sheath and positioned at right ventricular septum under fluoroscopy guidance [2, 3]. However, in superobese patients, venous access and fluoroscopy could potentially constitute a limit and lead to implant failure. Furthermore, some issues may also be related to telemetry for device interrogation. Indeed, the distance between device and the head of the programmer depends on orientation of antennas of device and programmer and in the worst case should be less than 10 cm. At present, no data is available about feasibility of Micra in superobese patients.

## 2. Case report

**Fig. 1.**
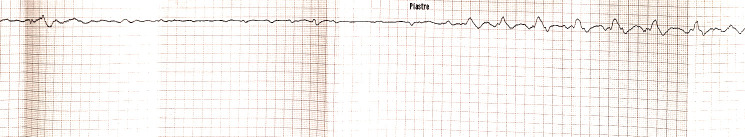
**Electrocardiogram strip showing asystole alternating to atrial fibrillation**.

A 70 years-old man was referred to our hospital for recurrent syncope. His medical history was positive for diabetes mellitus, arterial hypertension, dyslipidaemia and “super obesity”: he was 165 cm high and weighted 230 kg. Body mass index (BMI) was 84.4 kg/$m^{2}$. A dedicated bariatric ambulance was necessary for the transport to the hospital. At first medical contact, arterial blood pressure was 90/70 mmHg, heart rate 80 beats per minute, respiratory rate 18 breaths per minute, oxygen saturation 97%, blood glucose 231 mg/dL. The electrocardiogram documented a previously unreported atrial fibrillation (AF) with normal ventricular rate. During the observation in the Emergency Department, a cardiac arrest due to asystole occurred (Fig. 1). Cardiopulmonary resuscitation was immediately started with chest compressions and adrenaline administration and continued for five minutes, until the patient had a return of spontaneous circulation. He was then admitted to the Cardiac Intensive Care Unit during the night, where other asystole paroxysms continued in the subsequent minutes. A temporary pacemaker implantation was indicated in emergency, but fluoroscopy guidance was not possible because the bariatric bed precluded the use of the Cardiac Intensive Care Unit fluoroscope. The temporary pacemaker was successfully inserted through the right jugular vein into the right ventricle under ultrasound guidance. The echocardiogram (which was technically limited by the BMI of the patient), showed a preserved left ventricular ejection fraction, normal right ventricular function, dilation of both atria, absence of valvular disease, absence of pericardial effusion. Laboratory tests did not show any secondary cause of asystole episodes, but highlighted leukocytosis (white blood cells 22,670/μL), neutrophilia (19,180/μL), high C-reactive protein (4.24 mg/dL), renal insufficiency (serum creatinine 3.16 mg/dL). Thromboembolic risk was high (${\mathrm{CHA}}_{2}$${\mathrm{DS}}_{2}$VASc score = 3). During the subsequent hospital stay, the patient presented continue alternation of AF with high ventricular rate alternated to temporary pacemaker intervention. No secondary causes of asystole were found; therefore, a permanent pacing was definitely indicated in this patient.

A “heart team” discussion was done to decide the better care of the patient: a conventional pacemaker inserted percutaneous through venous access as cephalic, axillary or subclavian was judged highly challenging because of BMI of the patient. Obtaining a cephalic or axillary or even subclavian venous access with conventional technique is highly difficult in this kind of patient and the conventional leads may result too short to reach the right ventricle. Moreover, the presence of diabetes, temporary pacing, renal insufficiency, the need for oral anticoagulants and positive inflammatory indices in superobese patient increased the risk of infection of the device [4]. Therefore, conventional pacing procedure was discarded. The chosen option was leadless pacemaker implantation in a multidisciplinary intervention with surgical management of the femoral venous access. With an estimated battery longevity of 13.6 years, considering age and life expectancy of the patient, the leadless pacemaker was deemed appropriate [1].

**Fig. 2.**
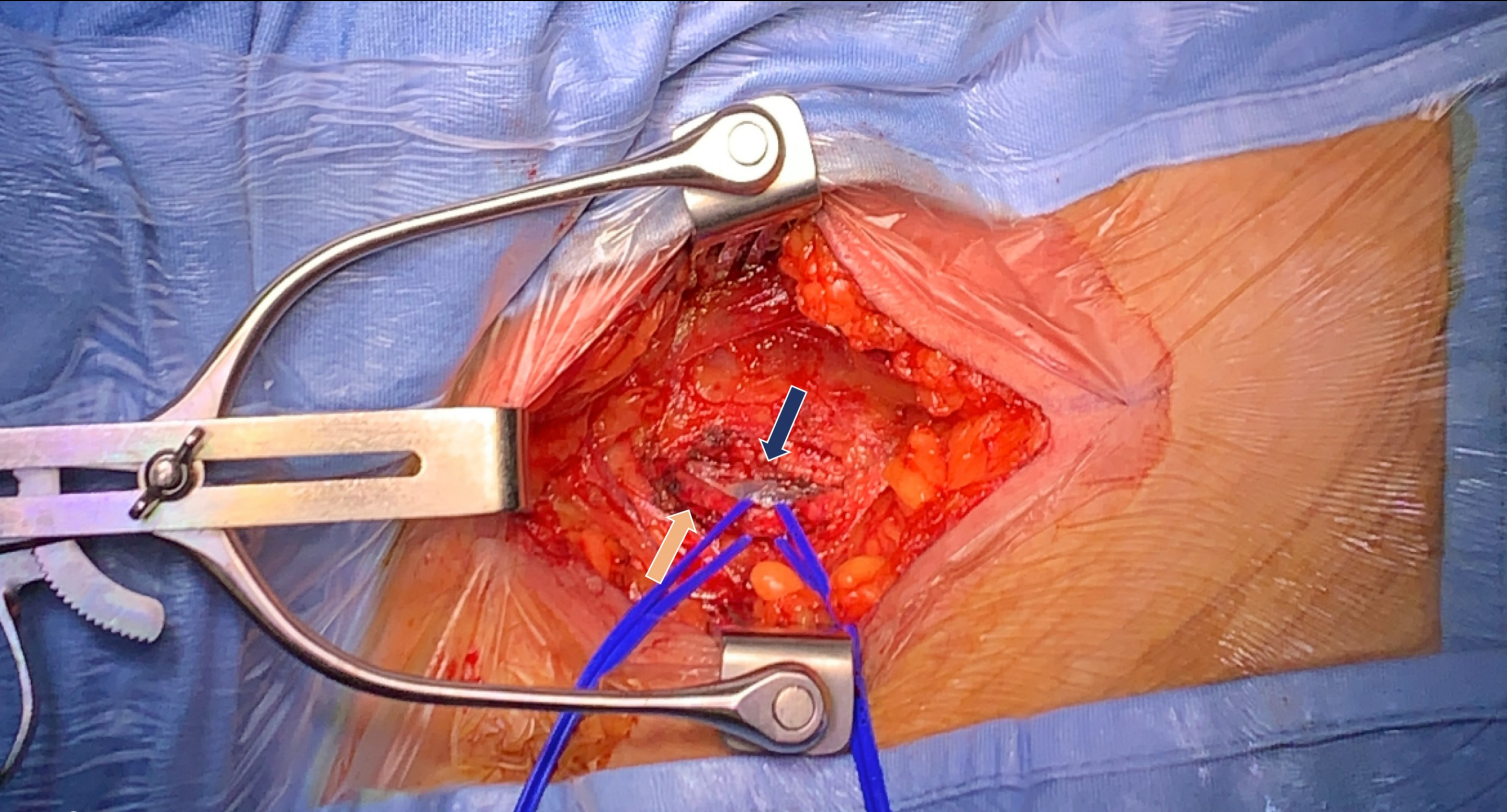
**Surgical isolation of femoral vein (blue arrow) and artery (orange arrow)**.

**Fig. 3.**
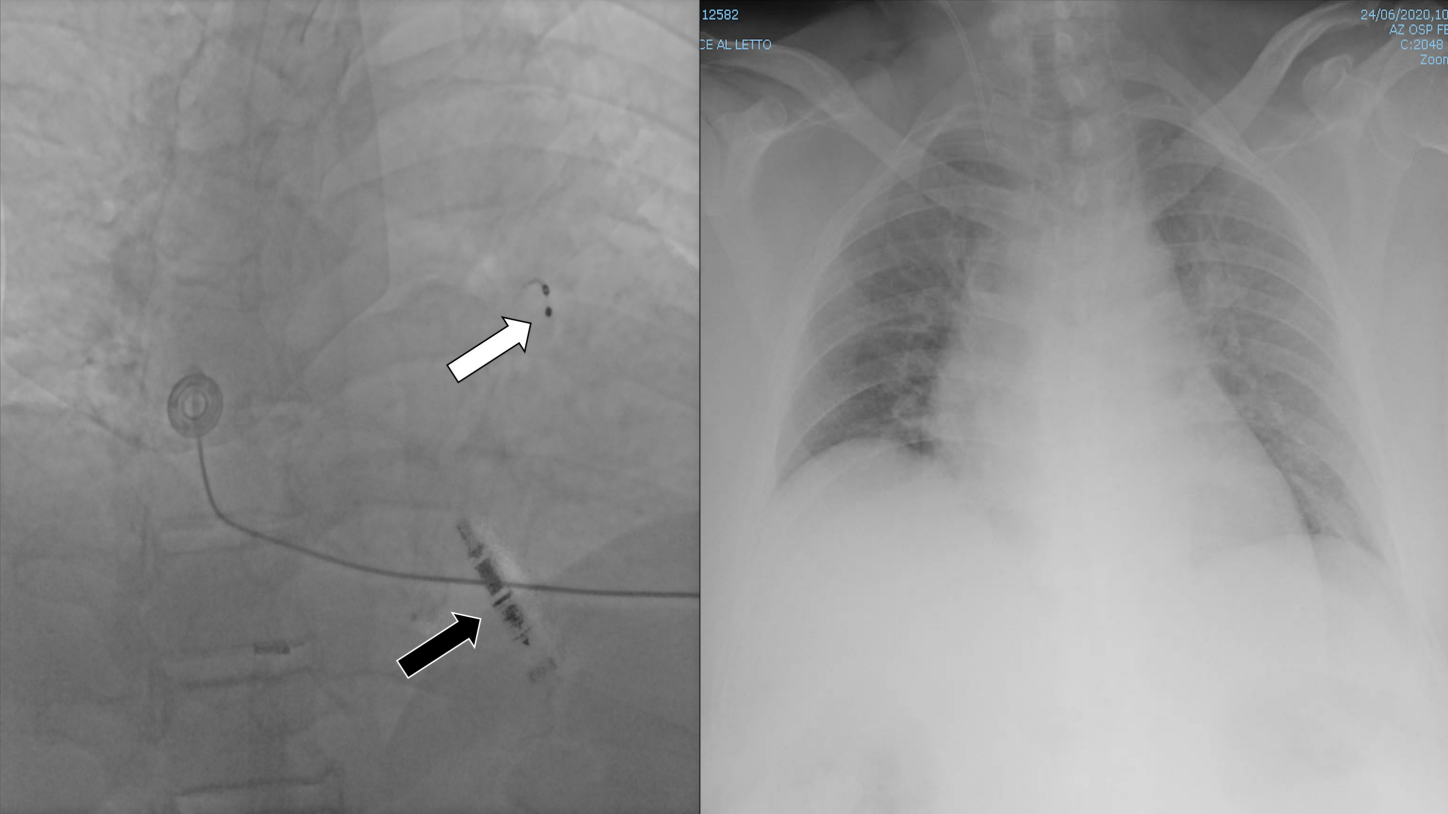
**Intraoperative fluoroscopy (left) and postoperative chest X-rays (right)**. Note the position of temporary pacemaker in right ventricular outflow tract (white arrow). Visualization of leadless pacemaker (and transcatheter delivery system) was of good quality with intraoperative fluoroscopy (left panel, black arrow) and of insufficient quality with chest X-rays (right panel).

The subsequent day, in a fully equipped operating room with bariatric table and appropriately dimensioned fluoroscope, a vascular surgeon performed surgical isolation of the right common femoral vein (Fig. 1). After that, two electrophysiologists proceeded to insert sheaths of increasing size until 23-Fr-internal/27-Fr-external via the femoral vein, and through that a steerable transcatheter delivery system for the device (Medtronic, Minneapolis, MN, USA). Fluoroscopic intraoperative images were of sufficient quality to guide the procedure (Fig. 2) and the Micra leadless pacemaker was inserted in the right ventricle and positioned in the interventricular septum. At that moment, it was observed that the temporary pacemaker was located at right ventricular outflow tract but it was pacing efficiently with acceptable electrical parameters. Therefore, it was decided to lease temporary pacing and during deployment and fixation of Micra to myocardial wall, particular attention was dedicated to not engage the temporary pacemaker in the fixation mechanism. After Micra deployment electrical parameters of sensing, pacing threshold and impedance were optimal. Vascular haemostasis and suture were performed by the vascular surgeon. No complications occurred. Postoperative imaging with X-rays performed in the ward was of poor quality and unable to show the implanted device (Fig. 3). Echocardiogram and device interrogation with dedicated programmer confirmed the good position and performance of Micra. The device was programmed in VVI mode with a lower rate of 60 bpm. After a few days, electrical control of the device confirmed optimal electrical parameters and the patient was discharged at home. One month after intervention device control confirmed optimal electrical parameters showing 35% of ventricular pacing.

## 3. Discussion

This case highlights safety and efficacy of leadless pacemaker implantation in a superobese patient.

In such a case, three aspects deserve consideration.

First, in superobese patients, fluoroscopy use is limited by the poor quality of the images. In our case, fluoroscopic guidance was useless for the positioning of temporary pacemaker in a cardiac intensive care unit setting and chest X-rays was scant for the visualization of the implanted permanent device. However, the Micra device was correctly visualized during the implantation and in such a setting intraoperative fluoroscopy was adequate for the correct positioning of the device.

Second, the vascular access is crucial for the success of the procedure and, if inadequate, could compromise the implantation of a device. Our case demonstrates that, even in a 230 kg patient, the femoral vein could be used for the insertion of the 23-Fr-internal/27-Fr-external introducer sheath that is required for the transcatheter delivery system. We observed that surgical isolation of the vein resulted in no complication. It is important to plan surgical isolation in a multidisciplinary team setting in such extreme cases.

Third, usually the device interrogation requires a distance <10 cm from the head of the programmer and the device, but this distance may vary depending on antennas’ orientation (signal is strong when antennas are parallel). Despite the BMI of this patient, device interrogation was feasible without issues.

Finally, based on these considerations when we face difficult implant like that in superobese, the option of leadless pacemaker must be considered. This option also reduces infectious risk in such high-risk patients.

## 4. Conclusions

Leadless pacemaker implantation can be effectively and safely performed even in superobese patients. Vascular access, fluoroscopic guidance and electronic interrogation could be easily managed and do not constitute a limit.
